# Circulating amino acids and cardiometabolic risk profile in offspring of women with type 1 diabetes: cross-sectional case-control study

**DOI:** 10.1038/s41598-025-07177-1

**Published:** 2025-07-01

**Authors:** Erik Somersalo, Hannu Kautiainen, Miira M. Klemetti, Johan G. Eriksson, Merja K. Laine

**Affiliations:** 1 Department of General Practice and Primary Health Care, University of Helsinki, Helsinki University Hospital, Helsinki, Finland; 2 Folkhälsan Research Center, Helsinki, Finland; 3 Primary Health Care Unit, Kuopio University Hospital, Kuopio, Finland; 4 Department of Obstetrics and Gynecology, University of Helsinki, Helsinki University Hospital, Helsinki, Finland; 5 Obstetrics and Gynecology, South Karelia Central Hospital, Lappeenranta, Finland; 6 Department of Obstetrics and Gynaecology and Human Potential Translational Research Programme, Yong Loo Lin School of Medicine, National University of Singapore, Singapore, Singapore

**Keywords:** Amino acids, Cardiometabolic disease, Offspring, Type 1 diabetes, Medical research, Risk factors

## Abstract

Branched-chain amino acids (BCAAs) are known to be associated with cardiovascular disease risk in adults. The aim of this study is to investigate whether an increased cardiometabolic risk profile can be observed in the amino acid profile of young adult offspring of women with type 1 diabetes. This cross-sectional case-control study included 73 offspring born to women with type 1 diabetes (cases) and 82 control participants (controls). At the age of 18–23 years, they participated in a clinical assessment including laboratory tests and questionnaires. Amino acid levels were analyzed from venous serum samples after 10 h of fasting using nuclear magnetic resonance (NMR) spectroscopy. No differences in cardiovascular disease or cardiometabolic risk factors were observed between the cases and the controls. Circulating amino acid levels were similar in both groups. The glucogenic score (combined alanine, glycine) was higher in overweight case men (case versus controls adjusted *p* = 0.015 (mean ratio 1.25 [95% CI 1.11 to 1.49]). The present findings do not support our hypothesis that serum amino acid profiles, determined in early adulthood, are associated with a more adverse cardiometabolic risk profile in offspring of women with type 1 diabetes. Further studies are warranted to clarify the potential role of amino acids in the development of cardiovascular disease in offspring of women with type 1 diabetes.

## Introduction

Cardiovascular diseases (CVD) are multifactorial, and healthy lifestyle choices can largely reduce the risk of developing CVD^1,2^. Well-known CVD risk factors include elevated blood pressure, hyperglycemia, dyslipidemia, as well as overweight and obesity^3^. However, more information on risk factors that characterize specific patient subgroups is needed to improve individualized primary and secondary prevention of CVDs^4,5^. Amino acids (AAs) play a key role in metabolic pathways^6^. They are involved in energy production and regulation, acting as signaling molecules influencing insulin sensitivity, lipid metabolism, and inflammation^7–10^. All these processes are linked to cardiometabolic risk factors such as obesity, hypertension and type 2 diabetes (T2D)^11,12^. The nuclear factor-κB (NF-κB) signaling pathway regulates immune responses and inflammatory processes^13^. Dysregulation of this pathway has been documented in chronic diseases, including type 1 diabetes (T1D)^13,14^. In a mouse model, diabetes during pregnancy has been shown to cause dysregulation of NF-κB in the mouse embryos^15^.

Nuclear magnetic resonance (NMR) is a spectroscopic technique used in metabolomics to identify metabolites, such as circulating lipids and AAs, on a larger scale^16^. Alterations in metabolic pathways can be detected and can be used to predict disease risk and potentially to guide therapy^17–19^. Elevated circulating levels of branched-chain amino acids (BCAAs) have been reported to be associated with obesity-related conditions such as insulin resistance, T2D, and CVD^20–23^. Five BCAAs and aromatic amino acids (AAAs), namely isoleucine, leucine, valine, tyrosine, and phenylalanine have shown highly significant associations with future development of T2D^19^. BCAAs are associated with obesity during childhood and adolescence and predict insulin resistance in this age group^24^. A Finnish study showed that circulating leucine and isoleucine levels are related to triglyceride levels in young women^25^. BCAAs regulate protein synthesis, cell growth, and autophagy, as well as insulin secretion through mammalian target of rapamycin (mTOR) activation^26–28^. Overstimulation of mTOR causes insulin resistance^29^. High concentrations of BCAAs have also been associated with increased production of reactive oxygen species and mitochondrial dysfunction, contributing to oxidative stress and inflammation^9^.

To the best of our knowledge, no previous studies have examined circulating AA concentrations in young adult offspring of women with T1D. Offspring of women with T1D have an increased risk of diabetes and early-onset CVD^30^. This can be explained by fetal programming: High blood glucose levels during pregnancy can alter fetal development, leading to long-term metabolic and vascular dysfunction^31,32^. Exposure to hyperglycemia can impair blood vessel function, increasing the risk of hypertension and atherosclerosis^33,34^. Maternal diabetes can influence gene expression through epigenetic changes, predisposing the child to metabolic and cardiovascular diseases later in life^35–37^. Offspring born to women with T1D are more likely to develop impaired glucose tolerance, insulin resistance, and eventually T2D, all of which increase the risk of CVD^38,39^. Studies suggest that offspring of women with T1D show early signs of arterial stiffness and endothelial dysfunction, which increase long-term CVD risk^40,41^. In addition, T1D during pregnancy is associated with altered lipid metabolism in offspring, predisposing them to CVD^42,43^.

Considering what is already known about BCAAs and the pathogenesis of CVD/T2D, we hypothesized that the AA profile of offspring of women with T1D would differ from that of offspring of women without diabetes.

The aim of this study was to investigate whether there are differences in the AA profile in early adulthood between offspring of women with T1D and offspring of women without diabetes, and whether this is associated with a more adverse cardiometabolic risk profile.

## Methods

### Design and participants

This study is a cross-sectional case-control study conducted in the Hospital District of Helsinki and Uusimaa, Finland. The study population included individuals born between January 1, 1996, and December 31, 2000, in the Helsinki metropolitan area.

The case group (*n* = 238) consisted of singleton offspring of women with T1D who were delivered by elective cesarean section at the Department of Obstetrics and Gynecology, Helsinki University Hospital, Finland. During the study period, all deliveries of women with T1D in the Hospital District of Helsinki and Uusimaa were centralized at this hospital.

The control group (*n* = 276) comprised of the first or second singleton child born after each case to women without diabetes within the same hospital district.

At 18–23 years of age, all participants were invited to take part in a clinical study and received an invitation letter. A total of 81 cases (36%) and 86 controls (20%) agreed to participate. Blood samples were available and analyzed in 75 cases and 82 controls. Two cases were excluded due to overt diabetes, resulting in a final sample size of 73 cases and 82 controls.

### Clinical study

The methods applied have been described previously^44^. All participants were examined by trained study nurses. Weight and body composition were measured in light indoor clothing without shoes or socks on using a bioimpedance body composition device (InBody 3.0, Biospace, Seoul, South Korea). Body weight and fat mass were recorded with an accuracy of 0.1 kg, and fat percentage was calculated. Height was measured without shoes or socks on against a wall-mounted stadiometer (SECA Telescopic measuring rod, SHZ, cm INT) with an accuracy of 0.1 cm. Body mass index (BMI) was calculated as kg/m^2^. Normal weight was defined as BMI < 25.0 kg/m^2^, and overweight was defined as BMI ≥25.0 kg/m^2^. Blood pressure was measured three times from the right arm in a sitting position after at least 15 min of rest using a cuff size 22 × 42 cm (Omron Intellisense M6 AC, Omron Healthcare Co. Ltd., Japan) and the mean value of the measurements was documented. Between the measurements there was at least a one-minute pause.

### Laboratory analyses, physical activity, and questionnaires

Venous blood samples were taken in a sitting position with a light stasis after 10 h of fasting. A 2-hour 75 g oral glucose tolerance test was performed according to the WHO 1999 guidelines. Fasting insulin, glycated hemoglobin A1c, total cholesterol, high-density lipoprotein cholesterol, low-density lipoprotein cholesterol, triglycerides, and high-sensitivity C-reactive protein were measured. Homeostatic Model Assessment of Insulin Resistance (HOMA-IR) was calculated.

Physical activity was determined using the Kuopio Ischaemic Heart Disease (KIHD) questionnaire and reported as Metabolic Equivalent of Task-hours per week (MET-h/week)^45^. The standardized questionnaire estimates physical activity during the previous 12-month period by type of physical activity, duration, and frequency. Each type of activity is assigned a MET-value. 1 MET = 3.5ml O_2_/kg/min, which roughly corresponds to the energy expenditure of metabolic state at rest. MET-h/week describes the sum of the product of each activity’s MET value, duration and frequency.

Smoking habits and chronic diseases were self-reported using validated questionnaires. Participants who reported hypertension, heart failure, arrhythmia, coronary heart disease or myocardial infarction, other heart disease, stroke, or cerebral hemorrhage were considered to have CVD.

### Metabolomics

Nightingale NMR was applied for the quantification of metabolite measures from serum samples. In this study, we focused on AAs, namely alanine, glycine, histidine, phenylalanine, tyrosine, leucine, isoleucine, and valine. We grouped the AAs, as done previously and according to Mikkola et al.^46^. Sum scores were created for glucogenic (GAA) (alanine and glycine), aromatic (AAA) (histidine, phenylalanine, and tyrosine), and BCAA (leucine, isoleucine, and valine).

### Statistics

Summary statistics are described using mean and standard deviation (SD), or numbers as percentages. Statistical evaluation between groups (case and controls) were analyzed by using Student’s t-test, Permutation test, Pearson’s chi-squared test, and Fisher’s exact test. Relationships between BMI groups (< 25.0 and ≥ 25.0) and case and controls were analyzed using permutation type two-way analysis of variance (ANOVA). In cases of violation of the assumptions (e.g., non-normality), a bootstraptype method was used for the continuous variables, and Monte Carlo p-values (small number of observations) were used for the categorical variables. The Stata 18.0 (StataCorp LP; College Station, Texas, USA) statistical package was used for the analysis.

## Results

No differences in characteristics were observed between cases and controls (Table 1). The mean age in both groups was 21 years. 59 (81%) of the cases and 61 (74%) of the controls were non-smokers. Physical activity was similar in both groups (30.9 MET-h/week [SD 28.5] versus 37.2 MET-h/week [SD 45.0]). Mean BMI was at the top of the normal reference range in both groups (24,7 kg/m^2^ [SD 5.0] among cases and 24,2 kg/m^2^ [SD 5.3] among controls). Body fat percentage was similar in both groups (33.0% [SD 7.8] versus 30.4% [SD 9.5] in women and 19.1% [SD 10.6] versus 17.4% [SD 7.1] in men). Mean blood pressure was within the normotensive range in both groups (118/74 mmHg among cases and 119/74 mmHg among controls). No differences between the two groups were observed in laboratory tests evaluating glucose metabolism. Mean lipid values were in the reference range for both groups, and no major differences were observed. 7% of cases and 5% of controls reported CVD.

**Table 1:** Clinical characteristics of the study participants (*N* = 155) divided into offspring of women with type 1 diabetes (cases) and offspring of women without diabetes (controls).

	Case *N* = 73	Controls *N* = 82	*P*-value
Women, *n* (%)	47 (64)	56 (68)	0.61
Age, years, mean (SD)	21 (2)	21 (2)	0.56
Smoking, *n* (%)			0.50
No	59 (81)	61 (74)	
Occasionally	9 (12)	11 (13)	
Current	5 (7)	10 (12)	
Physical activity, MET-h/week, mean (SD)	30.9 (28.5)	37.2 (45.0)	0.33
Body mass index (kg/m^2^), mean (SD)	24.7 (5.0)	24.2 (5.3)	0.60
Body fat percentage (%), mean (SD)			
Women	33.0 (7.8)	30.4 (9.5)	0.14
Men	19.1 (10.6)	17.4 (7.1)	0.51
Blood pressure (mmHg), mean (SD)			
Systolic	118 (12)	119 (10)	0.52
Diastolic	74 (9)	74 (7)	0.79
Plasma glucose (mmol/L), mean (SD)			
Fasting/0-h	5.38 (0.42)	5.38 (0.41)	0.99
2-h	5.83 (1.65)	5.63 (1.49)	0.44
Fasting serum insulin, mU/L, mean (SD)	11.2 (9.1)	10.9 (7.1)	0.83
Glycated hemoglobin A1c, mmol/mol, mean (SD)	33 (3)	33 (2)	0.67
Homeostatic Model Assessment of Insulin Resistance, mUxmmol/L^2^, mean, (SD)	2.74 (2.35)	2.65 (1.90)	0.79
Total cholesterol, mmol/L, mean (SD)	4.38 (0.74)	4.29 (0.64)	0.41
Low-density lipoprotein cholesterol, mmol/L, mean (SD)	2.58 (0.70)	2.53 (0.63)	0.58
High-density lipoprotein cholesterol, mmol/L, mean (SD)	1.59 (0.39)	1.56 (0.37)	0.65
Triglycerides, mmol/L, mean (SD)	0.91 (0.54)	0.97 (0.46)	0.49
High sensitivity C-reactive protein, mg/L, mean (SD)	2.41 (4.32)	3.03 (5.44)	0.44
Comorbidity, n (%)			
Asthma	9 (13)	10 (12)	0.93
Atopy	10 (14)	7 (9)	0.28
Psoriasis	3 (4)	1 (1)	0.34
Neurologic diseases	12 (17)	11 (13)	0.55
Cardiovascular disease	5 (7)	4 (5)	0.73
Rheumatic diseases	0 (0)	3 (4)	0.25

*MET* metabolic equivalent, *SD* standard deviation.

**Fig. 1:**
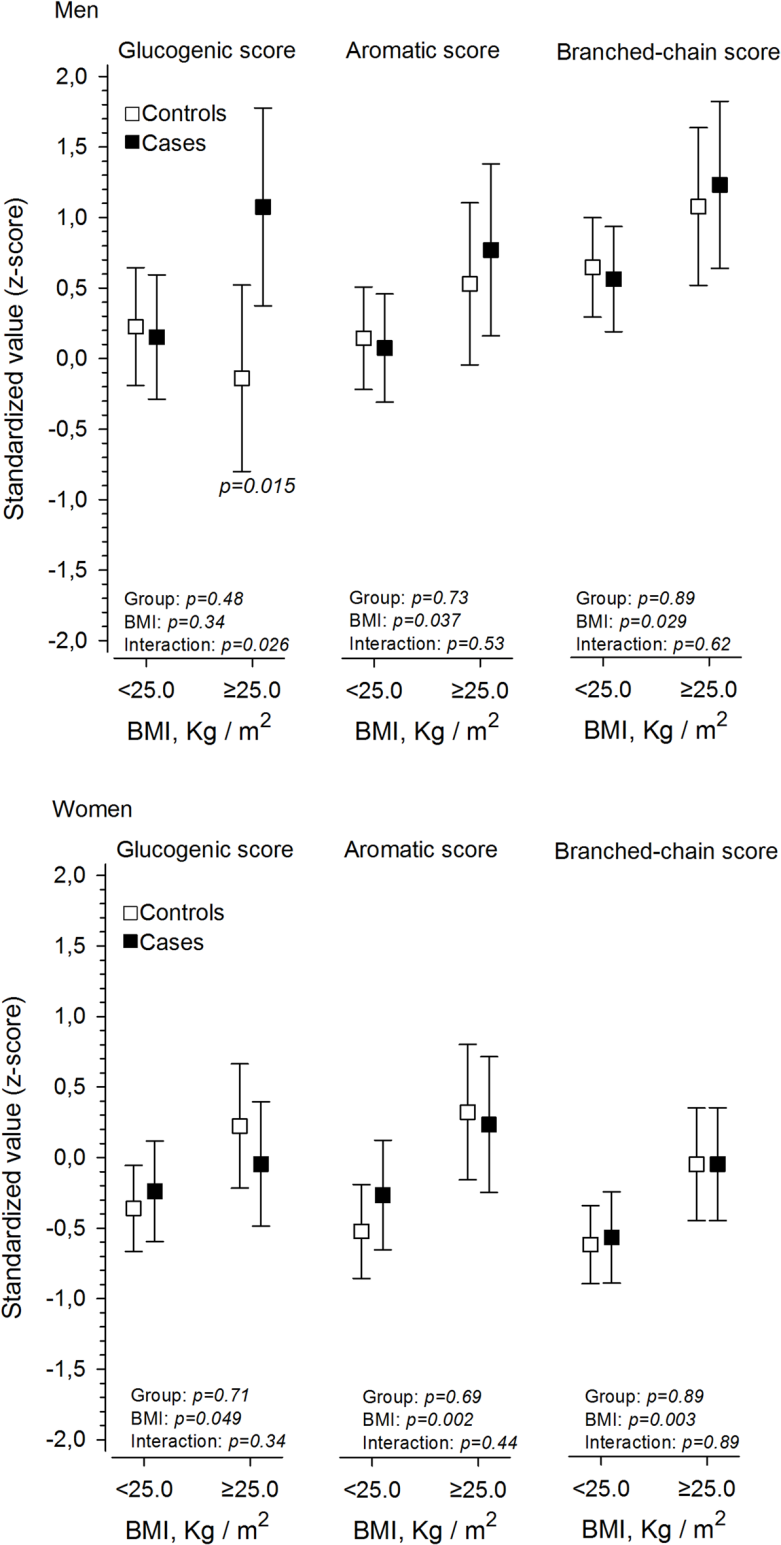
Adjusted (physical activity, fasting glucose and smoking) amino acid concentrations for men and women across body mass index (BMI) for offspring of women without diabetes (controls) and offspring of women with type 1 diabetes (cases). BMI $\:\ge\:$ 25.0 indicates overweight. Sum scores were created for glucogenic (alanine and glycine), aromatic (histidine, phenylalanine, and tyrosine), and branched chain (leucine, isoleucine, and valine) amino acids.

AA concentrations in cases and controls are reported separately for women and men in Table 2. No statistically significant differences were found between the two groups. Adjusted (physical activity, fasting glucose and smoking) amino acid concentrations across BMI for cases and controls are shown in Fig. 1. The glucogenic score was higher in overweight case men vs. overweight controls (*p* = 0.015 (mean ratio 1.25 [95% CI 1.11 to 1.49]).

**Table 2:** Amino acid concentrations of offspring of women with type 1 diabetes (cases) and offspring of women without diabetes (controls) reported separately for women and men.

Amino acid, mmol/L	Case *N* = 73	Controls *N* = 82	*P*-value	Mean Ratio (95% CI)
Number				
Women	47	56		
Men	26	26		
Alanine, mean (SD)				
Women	0.327 (0.055)	0.328 (0.082)	0.91	1.00 (0.91–1.08)
Men	0.342 (0.085)	0.323 (0.070)	0.39	1.06 (0.92–1.19)
Glycine, mean (SD)				
Women	0.230 (0.067)	0.228 (0.062)	0.88	1.01 (0.90–1.12)
Men	0.227 (0.048)	0.233 (0.057)	0.70	0.98 (0.85–1.10)
Histidine, mean (SD)				
Women	0.078 (0.010)	0.076 (0.011)	0.23	1.03 (0.98–1.09)
Men	0.079 (0.009)	0.081 (0.008)	0.42	0.98 (0.92–1.03)
Phenylalanine, mean (SD)				
Women	0.060 (0.010)	0.060 (0.014)	0.82	0.99 (0.91–1.07)
Men	0.060 (0.008)	0.061 (0.008)	0.43	0.97 (0.90–1.04)
Tyrosine, mean (SD)				
Women	0.053 (0.011)	0.051 (0.013)	0.54	1.03 (0.94–1.12)
Men	0.060 (0.013)	0.058 (0.009)	0.50	1.04 (0.93–1.14)
Leucine, mean (SD)				
Women	0.100 (0.022)	0.101 (0.023)	0.85	0.99 (0.91–1.08)
Men	0.126 (0.019)	0.131 (0.016)	0.31	0.96 (0.89–1.03)
Isoleucine, mean (SD)				
Women	0.045 (0.013)	0.046 (0.011)	0.79	0.99 (0.89–1.08)
Men	0.058 (0.012)	0.060 (0.009)	0.58	0.97 (0.88–1.07)
Valine, mean (SD)				
Women	0.199 (0.038)	0.197 (0.038)	0.99	1.01 (0.93–1.08)
Men	0.242 (0.036)	0.247 (0.033)	0.58	0.98 (0.90–1.05)

*SD* standard deviation.

Bootstrap type multivariate Hotelling’s T-squared test: *p* < 0.001.

Adjusted using Hommel’s method.

## Discussion

Our findings suggest that young adult offspring of women with T1D do not possess a distinctive cardiometabolic risk factor profile or widely deviant levels of circulating AAs potentially associated with CVD, compared to offspring of women without diabetes. The GAA score (a combination of alanine and glycine) was higher in overweight men born to women with T1D compared to men born to women without diabetes during pregnancy. The same phenomenon was not observed among overweight women. Among the eight AAs measured, no significant differences were observed between cases and controls. AA scores were BMI-dependent, with overweight status being associated with higher AA scores.

The use of GAA score is not well-established in research. In our case, this sum score was formed by the two AAs that did not belong to the BCAA or AAA category, which have been linked to CVD development^22^. The higher GAA score among overweight men born to women with T1D is surprising, since glycine has been inversely associated with BMI and CVD risk-factors, usually indicating favorable metabolic health^47–49^. Alanine has been linked to CVD risk factors including high blood pressure and insulin resistance, and therefore the results are expected^50,51^. In a Finnish study, in an age group significantly older than our study population, alanine was associated with coronary artery disease^52^.

The reason why the glucogenic score does not follow a similar pattern in men and women remains unknown. Hormonal factors may influence the results. Mikkola et al. investigated the association between body composition in older men and women and the same eight AAs and sum scores we considered in our study^46^. In their study, glucogenic score was associated with fat mass only in women. Alanine showed a positively association with fat mass in both women and men, while glycine was inversely associated with lean mass in men^46^. It remains speculative whether more subgroups with distinctive AA profiles could have been identified if there had been a bigger weight difference between cases and controls. Metabolic flexibility and AA levels are impacted by obesity^53,54^.

Since CVD typically manifests later in life, it can be assumed that the young age of the participants may have influenced the result^2^. AA levels are often age-dependent^55^. However, BCAAs have been associated with obesity and insulin resistance even in children^24,56^. In addition, some AAs have been shown to predict insulin resistance and T2D years in advance^19^. A recent study in adolescents with prediabetes or T2D identified a distinct AA profile, characterized by elevated levels of BCAAs and AAAs along with reduced glycine concentration^57^. Similar findings might have been expected in our study, given that offspring of women with T1D have an increased risk of T2D^39^. Our results are likely influenced by the overall good health of the participants. This may, in turn, be affected by selection bias, as those in better health may have been more likely to participate in the study.

Metabolomic measures are relatively dynamic and affected by intrinsic and extrinsic exposures^18^. This makes it difficult to define the clinical relevance of our study findings. In addition to the fact that most of the examined AAs are dietary essential, it is also known that the gut microbiome affects the bioavailability of AAs^58^. It would have been interesting to consider the participants’ diet, but, unfortunately, nutritional data was not collected in the present study. A follow-up study including dietary analysis could provide additional information on the effect of T1D during pregnancy on circulating AA levels in offspring. Diet should be considered holistically when examining cardiometabolic diseases. In addition to AAs, special attention should be paid to carbohydrates and fats^59,60^.

Moreover, our limited sample size might have led to some degree of selection bias, and the fact that the cohort included only participants from the Hospital District of Helsinki and Uusimaa, Finland, may reduce the generalizability of our findings. It would have been valuable to consider the characteristics of the mothers of the study participants, such as the age at onset of type 1 diabetes or the duration of the disease. However, these data were not available to us. In addition, it should be noted that the participants are relatively young in the context of CVD risk.

Despite its limitations, the present study is important considering our limited knowledge on the long-term metabolic impacts of in-utero exposure to maternal T1D. One key strength of our research is the thorough clinical examination of the participants, which reduces the likelihood of errors due to reporting bias. Although the NMR technique offers possibilities for more extensive metabolomic analyses, we decided to focus on the manageable entity of eight circulating AAs. In the future, using a more comprehensive GAA score could help detect more finite differences between the AA profiles of offspring born to women with T1D vs. offspring of women without diabetes.

## Conclusions

The present results do not support our hypothesis that serum AA profile, determined in early adulthood, is associated with a more adverse CVD risk profile in offspring of women with T1D. However, the GAA score was higher in overweight men born to women with diabetes during pregnancy, compared to men born to women without diabetes during pregnancy. Further studies should be performed to find out whether these AAs could serve as early biomarkers of CVD development or whether changes in the AA levels over years could be used to monitor CVD progression in offspring of women with T1D.

## Data Availability

Due to legal restrictions, the data cannot be shared publicly; however, requests for access can be directed to the corresponding author.

## Abbreviations

AA: Amino acid
AAA: Aromatic amino acid
BCAA: Branched chain amino acid
CVD: Cardiovascular disease
GAA: Glucogenic amino acid
mTOR: Mammalian target of rapamycin
NF-κB: Nuclear factor-κB
NMR: Nuclear magnetic resonance
T1D: Type 1 diabetes
T2D: Type 2 diabetes
